# Secular trends in cardiovascular risk factors among women aged 45–54 years in Gothenburg, Sweden, from 1980 to 2014

**DOI:** 10.1186/s12889-020-09098-2

**Published:** 2020-07-01

**Authors:** Carina U. Persson, Anna-Clara Collén, Annika Rosengren, Zacharias Mandalenakis, Tatiana Zverkova Sandström, Michael Fu, Mikael Dellborg, Saga Johansson, Per-Olof Hansson

**Affiliations:** 1 Department of Clinical Neuroscience, Rehabilitation Medicine, Institute of Neuroscience and Physiology, Sahlgrenska Academy, University of Gothenburg, Gothenburg, Sweden; 2 Region Västra Götaland, Department of Physiotherapy, Sahlgrenska University Hospital/Östra, Gothenburg, Sweden; 3 Department of Molecular and Clinical Medicine, Institute of Medicine, Sahlgrenska Academy, University of Gothenburg, Gothenburg, Sweden; 4 Region Västra Götaland, Department of Medicine Geriatric and Emergency, Sahlgrenska University Hospital, Gothenburg, Sweden

**Keywords:** Epidemiology, Obesity, Physical activity, Population health

## Abstract

**Background:**

A declining trend in mean cholesterol levels and smoking has been observed in high-income western countries during the last few decades, whereas obesity rates have increased. Simultaneously, mortality from coronary heart disease has decreased. The aim of the present study was to determine whether the trends in cardiovascular risk factors have continued in successive cohorts of middle-aged women over a period of 34 years.

**Methods:**

Six population-based, cross-sectional samples of women (*n* = 2294) mean age: 49.8 years (range: 45–54), living in Gothenburg, Sweden, were investigated between 1980 and 2014.

**Results:**

Body mass index (BMI) increased over time, with a mean BMI of 24.7 kg/m^2^ in 1980 to 25.7 kg/m^2^ in 2013–2014, corresponding to a weight gain of 4.5 kg, together with an increase in the proportion of obese individuals (BMI ≥ 30 kg/m^2^) from 10.4 to 16.6% (*p* = 0.0012). The proportion of smokers and women with hypertension decreased from 34.5 to 12.8% (*p* = 0.0006) and from 37.7 to 24.5% (*p* < 0.0001) respectively. Mean total serum cholesterol levels decreased from 6.23 (SD 1.09) mmol/L in 1980 to 5.43 (SD 0.98) mmol/L in 2013–2014 (*p* < 0.0001). Self-reported leisure time regular exercise increased from 7.8% in 1980 to 35.6% in 2013–2014 (*p* < 0.0001). For women born in 1963, the prevalence ratio of not having any of five major cardiovascular risk factors was 1.82 (95% confidence interval (CI) 1.38–2.41), compared with women born in 1925–1934.

**Conclusion:**

The trend towards increasing obesity, more leisure-time physical activity and less smoking remains, while the decrease in serum cholesterol appears to have abated.

## Background

Since the 1970s, declining mortality trends in coronary heart disease have been observed inside and outside Europe, [1, 2] due mainly to better treatment and improvements in cardiovascular risk factors, [3, 4] predominantly lower smoking rates, serum cholesterol and blood pressure. While the incidence of fatal myocardial infarction has decreased in both sexes, some data suggest that the decline in non-fatal myocardial infarction may be less pronounced in middle-aged and younger women than in men of the same age [5]. Although risk factor patterns are improving and coronary heart disease mortality rates are decreasing in the western world, the opposite is occurring in the eastern parts of the world, such as China [6] and Eastern Europe [7].

In health promotion work, it is important for health-care providers and society to be aware of changes in risk factors over time. Few studies have provided information on secular trends in cardiovascular risk factors over an extended period [8–12]. We have previously reported a decline in cardiovascular risk factors in five cohorts of middle-aged women over a 23-year period, demonstrating lower rates of smoking, decreasing total serum cholesterol levels, fewer women with hypertension and an increase in leisure-time physical activity over time [13]. On the other hand, rates of obesity, triglyceride levels and experience of permanent stress increased during this period [13]. The same secular trends in cardiovascular risk factor patterns were seen among middle-aged men from the same geographical area from the 1960s until 2013 [12].

The aim of the present study was to determine whether the trends in predefined cardiovascular risk factors, obesity, hypertension, hypercholesterolemia, diabetes or smoking among middle-aged women have been maintained over time, by adding a sixth cohort of randomly selected middle-aged women, living in the same geographical area, and investigated using the same methodology as for the previous five cohorts.

## Methods

### Study population

The study population comprised 2294 women from six randomly selected cohorts of middle-aged women, all living in the city of Gothenburg, Sweden, who, between 1980 and 2014, participated in investigations (Table 1). Data on cardiovascular and coronary heart disease risk factors were collected using standardised questionnaires and physical examinations.

**Table 1:** Participation rates in six cohorts of middle-aged women living in Gothenburg, Sweden

Birth year	Year of examination	Age at examination, years	Number invited	Number examined	Participation rate (%)
1925–1934	1980	45–54	754	618	82
1931–1940	1985	45–54	246	207	84
1936–1945	1990	45–54	291	218	75
1941–1950	1995	45–54	354	241	68
1953	2003–2004	50–51	994	667	67
1963	2013–2014	50	645	343	53

Cohort 1 consists of a sample of women, born in 1925–1934 and aged 45–54 years when investigated between November 1979 and February 1981, from the Gothenburg BEDA study [14]. In this study, a random population sample of women living in the city of Gothenburg was invited to a screening examination of cardiovascular disease [14].

Cohorts 2, 3 and 4 consist of randomly sampled women aged 45 to 54 years participating in the WHO MONICA project, GOT-MONICA, [15, 16] screened in 1985, 1990 and 1995.

Cohort 5 derives from “The study of men and women born in 1953” [17]. One third of all women born in 1953 and living in the city of Gothenburg were randomly selected and invited to a health examination at the age of 50.

Secular trends in risk factor patterns in these five cohorts have previously been presented [13]. The new cohort (cohort 6) derives from “The study of men and women born in 1963” [18]. Every fifth woman born in 1963 and living in the city of Gothenburg was randomly selected and invited to participate in the study. The investigations were performed between October 2013 and April 2014, with all participants at the age of 50 years [18].

### Data collection and examination procedures

Data on smoking habits, leisure-time physical activity, mental stress, previous diseases and pharmacological treatments were obtained by questionnaires. Height was recorded to the nearest centimetre. Body weight was measured on a lever balance to the nearest 0.1 kg with the women barefoot and wearing light indoor clothing. Waist circumference was measured in cm at the level of the umbilicus. Body mass index (BMI, weight in kg/(height in m)^2^) was used as an indicator of overweight and obesity. Overweight was defined as a BMI of 25 kg/m^2^ or higher, while obesity was defined as a BMI of ≥30 kg/m^2^.

Blood pressure was measured, using a mercury manometer, in a seated position after at least 5 min’ rest and before venpuncture. Hypertension was defined as systolic blood pressure of > 140 mmHg and/or diastolic blood pressure of > 90 mmHg and/or treatment with antihypertensive agents.

Blood samples were drawn from the antecubital vein. Analyses of total serum cholesterol and triglyceride measurements were determined according to standard laboratory procedures. In cohorts 1–5, all the women were investigated in the morning after an overnight fast. In Cohort 6, for practical reasons, almost one third of the women were investigated in the afternoon after at least 4 h of fasting.

The diagnosis of diabetes mellitus was self-reported in the questionnaire. The women were classified as never smokers, former smokers or current smokers. The category of current smokers includes women who had quit smoking less than 1 month before the investigation.

Leisure-time level was assessed using the Saltin-Grimby Physical Activity Level Scale (SGPALS) [19]. The SGPALS is an ordinal scale comprising four response categories coded as: 1 = sedentary (physically inactive); 2 = some light physical activity, such as walking, riding a bicycle and light gardening for at least 4 h a week; 3 = regular moderate physical activity for a minimum of 3 h a week and 4 = regular hard physical training for competition sports. In the analyses, categories 3 and 4 were combined, due to the small number of women in category 4.

Mental stress was defined as feeling tense, irritable or filled with anxiety or having sleeping difficulties because of conditions at work or at home. The mental stress questionnaire comprises six response categories: 1 = never experienced stress; 2 = one period of stress ever; 3 = some periods of stress during the past 5 years; 4 = several periods of stress during the past 5 years; 5 = permanent stress during the past year and 6 = permanent stress during the past 5 years. Permanent stress was defined by category 5 or 6 [20].

The study complies with the Declaration of Helsinki.

### Statistical methods

The statistical analysis, data management and graphical presentation were conducted in SAS 9.3 (SAS Institute, Gary, North Caroline, USA). Each cohort was assigned a sequential number from 1 to 6, related to the order of the year of birth. The descriptive results are presented as a mean (standard deviation) for continuous data and as a frequency (percentage) for categorical data. One-way ANOVA was used to compare group means and the Cochran-Mantel-Haenszel test was used to examine differences in proportions, when a linear association between study groups was hypothesised. Equality of proportions was assessed with the χ^2^ test. The presence of trends over time in dichotomous variables was assessed using the Cochran-Armitage Asymptotic test. The mean change across the study cohorts was tested by linear regression for continuous variables and by logistic regression for categorical data. The results are presented as parameter estimates (95% CI). Prevalence ratios (risk ratios) of the number of risk factors were calculated with Cohort 1 as reference. Cohorts 5 and 6 were then further compared as follows: t-test for mean values; Wilcoxon’s non-parametric test for medians; the Mantel-Haenszel test for categorical and χ^2^ for dichotomous variables. The significance level was set at a *p*-value of < 0.05.

## Results

As shown in Table 1, the participation rates decreased, from over 80% in the first two cohorts, to 53% in the latest cohort. Table 2 shows anthropometric measurements, cardiovascular risk factors, physical activity levels and permanent stress in all six cohorts of women. From the first investigation in 1980 to the last in 2013–2014, the mean height and body weight increased by about two centimetres to 165.9 cm (*P* for trend < 0.0001) and 4.5 kg to 70.7 kg (*P* for trend < 0.0001) respectively. The mean waist circumference increased markedly; from 80.1 cm in 1985 to 88.4 cm in 2013–2014 (waist circumference was not measured in the first cohort). The proportion of obese women increased from 10.4 to 16.6% from 1980 to 2013. Throughout the same period, there was a trend towards a lower prevalence of hypertension.

**Table 2:** Secular trends in cardiovascular risk factors in six cohorts of middle-aged women

	Cohort 1 Born 1925–1934 Year of examination 1980	Cohort 2 Born 1931–1940 Year of examination 1985	Cohort 3 Born 1936–1945 Year of examination 1990	Cohort 4 Born 1941–1950 Year of examination 1995	Cohort 5 Born 1953 Year of examination 2003–2004	Cohort 6 Born 1963 Year of examination 2013–2014	*P* for trend	*P* for Cohort 5 versus cohort 6	Mean change between the studied cohorts	*P* for mean change
Variables	*N* = 618	*N* = 207	*N* = 218	*N* = 241	*N* = 667	*N* = 343				
Age, mean (SD)	49.5 (2.9)	49.8 (2.9)	49.1 (2.6)	49.7 (2.5)	50.2 (0.4)	50.0 (.0)				
Age, median (Q1; Q3)	49 (47; 52)	50 (47; 52)	49 (47; 51)	50 (48; 51)	50 (50; 50)	50 (50; 50)				
Height, cm	163.8 (5.8)	164.9 (6.2)	165.4 (6.1)	166.0 (6.4)	166.0 (6.8)	165.9 (6.6)	< 0.0001	n.s.	0.40 (0.10–0.70)	0.020
Weight, kg	66.2 (10.8)	65.9 (11.3)	68.1 (12.7)	69.2 (11.9)	70.5 (13.0)	70.7 (14.0)	< 0.0001	n.s.	1.06 (0.66–1.47)	0.0019
Waist circumference, cm	–	80.1 (10.7)	77.9 (10.4)	80.7 (10.3)	83.1 (11.4)	88.4 (11.7)	< 0.0001	<.0001	2.20 (−0.17; 4.57)	0.060
BMI, kg/m^2^	24.7 (3.9)	24.3 (4.0)	24.9 (4.5)	25.1 (4.2)	25.6 (4.5)	25.7 (4.9)	< 0.0001	n.s	0.27 (0.09–0.44)	0.014
BMI four categories, % (n)							0.0012	n.s		
BMI < 20	4.7 (29)	11.1 (23)	6.9 (15)	7.9 (19)	5.0 (33)	7.3 (25)			1.01 (0.93–1.11)	0.78
20 ≤ BMI < 25	55.5 (343)	54.6 (113)	55.1 (120)	49.0 (118)	49.2 (328)	47.2 (162)			0.93 (0.89–0.98)	0.0021
25 ≤ BMI < 30	29.5 (182)	25.6 (53)	25.7 (56)	32.0 (77)	30.7 (205)	28.9 (99)			1.02 (0.97–1.07)	0.51
BMI ≥ 30	10.4 (64)	8.7 (18)	12.4 (27)	11.2 (27)	15.1 (101)	16.6 (57)			1.13 (1.05–1.21)	0.0005
Systolic BP, mmHg, mean (SD)	135.3 (20.7)	128.7 (18.5)	125.5 (17.0)	128.4 (17.9)	123.1 (19.0)	129.3 (16.5)	< 0.0001	<.0001	−1.25 (−3.75; 1.24)	0.24
Diastolic BP, mmHg, mean (SD)	85.1 (11.1)	81.0 (9.4)	80.2 (9.3)	82.9 (9.9)	82.6 (10.8)	81.6 (9.8)	< 0.0001	n.s	−0.29 (−1.5; 0.92)	0.55
Blood pressure treatment, % (n)	9.71 (60)	6.28 (13)	10.55 (23)	7.88 (19)	9.60 (64)	7.62 (26)	n.s.	n.s.	0.98 (0.91–1.06)	0.66
Hypertension, % (n)	37.7 (233)	29.5 (61)	25.7 (56)	30.3 (73)	29.7 (198)	24.5 (84)	< 0.0001	0.081	0.91 (0.87–0.95)	< 0.0001
Serum cholesterol, mmol/L	6.23 (1.09)	6.23 (1.21)	5.82 (1.15)	5.60 (1.08)	5.44 (0.93)	5.43 (0.98)	< 0.0001	n.s.	−0.19 (−0.26; −0.11)	0.0025
Serum triglycerides, mmol/L	1.07 (0.54)	1.17 (0.68)	1.27 (0.51)	1.38 (0.75)	1.24 (1.14)	1.05 (0.67)	< 0.0001	0.0007	0.01 (−0.09; 0.10)	0.87
Self-reported diabetes, % (n)	1.78 (11)	2.42 (5)	0.92 (2)	2.07 (5)	1.95 (13)	1.17 (4)	n.s.	n.s.	0.97 (0.82–1.15)	0.72
Smoking habits, % (n)							0.0006	0.0006		
Never smoked	51.7 (319)	44.9 (92)	53.2 (116)	45.2 (109)	37.5 (250)	58.6 (201)			0.97 (0.93–1.01)	0.13
Former smokers	13.8 (85)	20.0 (41)	15.6 (34)	29.5 (71)	36.3 (242)	28.6 (98)			1.28 (1.21–1.35)	< 0.0001
Current smokers, 1–14 g/day	22.2 (137)	13.7 (28)	11.9 (26)	8.7 (21)	16.4 (109)	7.6 (26)			0.86 (0.81–0.91)	< 0.0001
Current smokers, > 14 g/day	12.3 (76)	21.5 (44)	19.3 (42)	16.6 (40)	9.8 (65)	5.2 (18)			0.88 (0.82–0.94)	< 0.0001
Physical activity in leisure time, % (n)							< 0.0001	0.0003		
Sedentary	23.5 (145)	19.5 (40)	13.5 (29)	28.6 (50)	13.8 (92)	10.5 (36)			0.85 (0.80–0.90)	< 0.0001
Light PA	68.7 (424)	73.2 (150)	75.8 (163)	56.0 (98)	62.3 (415)	53.9 (185)			0.88 (0.84–0.92)	< 0.0001
Regular PA	7.8 (48)	7.3 (15)	10.7 (23)	15.4 (27)	23.9 (159)	35.6 (122)			1.49 (1.39–1.60)	< 0.0001
Permanent stress, % (n)	16.4 (101)	16.1 (33)	19.5 (42)	17.0 (25)	22.8 (151)	22.2 (76)	0.0016	n.s.	1.09 (1.03–1.16)	0.0017
Born in Sweden, % (n)	86.57 (535)			81.33 (196)	76.61 (511)	75.22 (258)	< 0.0001	n.s.	0.85 (0.80–0.91)	< 0.0001

Continuous variables are presented as the mean (SD). Proportions are presented as percentages (number). *BMI* Body mass index;

*BP* Blood pressure

Waist data were missing in the first cohort and for another 10 women, a total of 628 women

Body mass index data were missing for nine women

Systolic blood pressure data were missing for 21 women

Diastolic blood pressure data were missing for 29 women

Triglyceride data were missing for 28 women

A diabetes history was missing for two women

A history of smoking was missing for four women

A history of physical activity level during leisure time was missing for 73 women

Data on self-perceived stress were missing for 106 women

Mean serum cholesterol decreased from 6.23 (SD 1.09) mmol/L in 1980 to 5.43 (SD 0.98) mmol/L in 2013–2014. The proportion of women with self-reported diabetes mellitus was low, ranging between 1.0 and 2.4%, without any significant change over time. The decrease in smoking has continued and, in the latest cohort, 13% of the women were current smokers, in contrast to 34.5% registered in the first cohort. High-intensity leisure-time physical activity has steadily increased over the decades. In the latest cohort, 35.6% of the women reported being physically active at an intensive level on a regular basis. Simultaneously, the proportion of women with a sedentary lifestyle declined from 23.5 to 10.5%. Self-reported permanent stress has increased over the years, but, in the latest cohort, women born in 1963, no further increase was found compared with women born in 1953.

When comparing only the latest two cohorts, a significant increase was observed in waist circumference, systolic blood pressure and physical activity during leisure time, while there was a significant decrease in s-triglycerides and the number of smokers (Table 2).

**Fig. 1:**
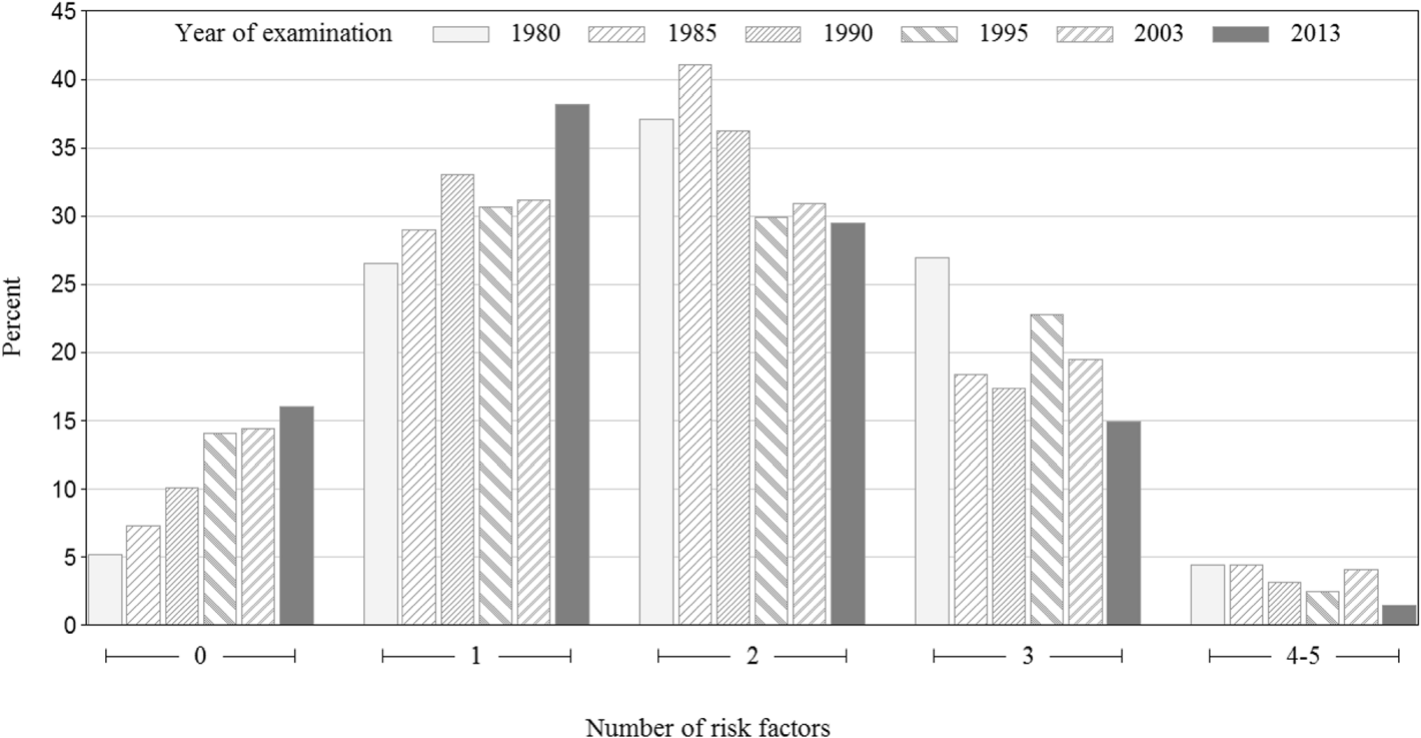
Percentage of participants with 0, 1, 2, 3 or 4–5 of 5 predefined cardiovascular risk factors in six cohorts of middle-aged women examined in: 1980, 1985, 1990, 1995, 2003–2004 and 2013–2014

Table 3 shows the prevalence ratios (PRs) for having none and one to four or five of the predefined cardiovascular risk factors (body mass index ≥25, hypertension, serum cholesterol > 5 mmol/L, diabetes and smoking), with women born in 1925–1934 (Cohort 1) as a reference. For women born in 1963, the PR for not having any of the predefined risk factors was 1.82 (95% confidence interval (CI) 1.38–2.41), while the PR for having four or five risk factors was 0.75 (95% CI 0.64–0.88). The percentage of participants with 0, 1, 2, 3 or 4–5 of the predefined risk factors is presented in Fig. 1.

**Table 3:** Prevalence ratios (PRs) for the number of predefined cardiovascular risk factors in six cohorts of middle-aged women

	O risk factor			1 risk factor			2 risk factors			3 risk factors			4 risk factors		
Year of birth	n	PR	95% CI	n	PR	95% CI	n	PR	95% CI	n	PR	95% CI	n	PR	95% CI
1925–1934	32	1.00		164	1.00		229	1.00		166	1.00		27	1.00	
1931–1940	15	1.11	0.91–1.35	60	1.03	0.94–1.13	85	1.04	0.96–1.13	38	0.89	0.82–0.97	9	1.00	0.82–1.21
1936–1945	22	1.26	1.01–1.58	72	1.09	0.99–1.20	79	0.99	0.91–1.08	38	0.88	0.81–0.95	7	0.93	0.78–1.11
1941–1950	34	1.52	1.18–1.96	74	1.06	0.96–1.17	72	0.92	0.84–1.00	55	0.94	0.86–1.03	6	0.87	0.74–1.03
1953	96	2.03	1.49–2.75	208	1.13	0.99–1.29	206	0.87	0.77–0.98	130	0.81	0.72–0.92	27	0.96	0.73–1.26
1963	55	1.82	1.38–2.41	131	1.23	1.09–1.37	101	0.89	0.81–0.98	51	0.79	0.72–0.87	5	0.75	0.64–0.88

Cardiovascular risk factors: body mass index ≥25, hypertension, serum cholesterol > 5 mmol/L, diabetes and smoking

Women born in 1925–1934 served as the reference group. In all, there were 36 women with missing data. *CI* Confidence interval; *PR* Prevalence ratio

**Fig. 2:**
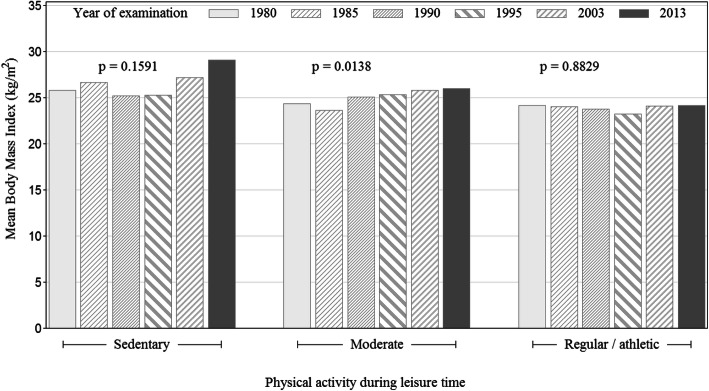
Mean body mass index (MBI) (x-axis) in six cohorts of middle-aged women with different levels of physical activity during leisure time (Y-axis)

The women who reported a regular/athletic physical activity level did not increase their mean BMI over time and nor did sedentary women. However, in those reporting moderate leisure-time physical activity levels, BMI increased significantly in successive cohorts (Fig. 2).

## Discussion

In the latest studied cohort, the trend towards increasing obesity, more leisure-time physical activity and less smoking remains, while the decrease in total serum cholesterol appears to have abated, with essentially identical mean levels in the two last cohorts. Since 1980, the prevalence ratio for not having any of the five predefined cardiovascular risk factors was more than 80% higher, while the prevalence ratio for having four or five of these risk factors decreased by 25%. These results are consistent with previously reported secular trends for men in the same region [12].

The pattern of reduced smoking and increased BMI is also similar to the results of the MONICA study from northern Sweden [9]. Likewise, in line with our results, an upward trend for regular exercise or athletic training during leisure time and a decreasing trend for smoking, blood pressure and cholesterol were observed in cross-sectional surveys of 40- to 42-year-old Norwegians from 1975 to 2010 [21].

In all six cohorts, the prevalence of diabetes mellitus was low, reflecting the general, comparatively low prevalence in the Swedish population [22]. The lack of an increase in diabetes, in spite of increasing obesity, has also been documented in some other European countries; the reason for this is not clear [23].

Even a minor increase in blood pressure levels in a large population may lead to marked increases in the burden of cardiovascular disease in the community [24]. In the current study, however, the blood pressure levels decreased between the first and the second cohort and have since then been comparatively stable. Due to the strong correlation between obesity and hypertension, increasing blood pressure levels and the prevalence of hypertension in the population are likely to occur in the future and may lead to an increase in cardiovascular diseases, especially stroke. A decreasing trend in the incidence of ischemic stroke in Sweden has been documented, however, with a worrying trend towards an increasing incidence in the young [25].

The trend towards an increasing level of leisure-time physical activity has also been observed in other countries such as Finland, Scotland, the USA, Canada, Taiwan and Australia [26]. A cross-sectional study of 2605 middle-aged Swiss men and women showed that a high level of physical activity was associated with a favourable cardiovascular risk profile. This was seen, even if the activity was only concentrated at weekends or was concomitant with an otherwise highly sedentary lifestyle [27].

In spite of the positive trend towards fewer smokers, they still account for 13% of the population. People who smoke often have other cardiovascular risk factors and a study of Norwegian adults showed that smokers were more likely to have low leisure-time physical activity levels compared with non-smokers [28].

Mental stress may act as a trigger for major cardiac events and influence the prognosis of cardiovascular disease and the progress of stress cardiomyopathy [29]. The same questionnaire for mental stress was used in a prior study of 6935 men aged 47 to 54 years at baseline without previous myocardial infarction [20]. That study found that permanent stress predicted non-fatal myocardial infarction or death from coronary artery disease during a mean follow-up of 11.8 years. In another study, based on a 37-year follow-up of middle-aged women in Gothenburg, the perception of high mental stress was associated with smoking and thereby a higher cardiovascular risk [30].

The trend towards declining serum cholesterol levels over time did not continue between 2003 and 2013–2014. The same trend was observed among Swedish men from the same geographical area [12]. In the Swedish Västerbotten County Study, however, the investigators found a trend towards increasing serum cholesterol levels, believed to be due to a higher consumption of saturated fat [11].

The main strength of this study is the well-defined population samples of women living in the same geographical area and examined at the same age with the same methods over three decades.

However, there are also some potential limitations to be considered. First, the cohort sizes were relatively small. Second, there is a risk of selection bias with decreasing participation rates, as participating women may differ from non-participating women. Participants in population studies tend to have a higher socioeconomic status and to be healthier than non-participants [31]. Accordingly, there is a risk of overestimating the positive changes that are seen, in addition to underestimating the adverse changes for corresponding reasons. The reduced participation rates in population studies are a well-known problem when comparing secular changes [32]. Third, the use of the Saltin-Grimby scale might have biased the results due to social desirability and recall bias, compared with measurements that are more objective [33]. The fact that several variables, such as smoking, diabetes, mental stress and physical activity, were self-reported can cause validity problems. In cohort 6, almost one third of the women were investigated in the afternoon after at least 4 h of fasting. The shorter period of fasting may influence the levels of triglycerides, while only a minor influence on serum-cholesterol levels is related to fasting [34]. The low prevalence of diabetes may be due to self-report bias related to diabetes status. In addition, the results cannot be generalised to other ages, parts of the world or to men. Cardiovascular risk factors are constantly changing as lifestyles are modified. As a result, new studies and updated data are needed from different parts of the world.

## Conclusions

The trend towards increasing obesity, more leisure-time physical activity and less smoking remains, while the decrease in serum cholesterol appears to have abated. Compared with 1980, the prevalence ratio for not having any of the five major cardiovascular risk factors was almost twice as high in 2013–2014. In spite of this, with one in seven women still smoking, one in six obese and a markedly increasing waist circumference, continuing efforts are necessary to improve health literacy and lifestyle changes, particularly in relation to preventing obesity.

## Data Availability

The datasets analysed during the current study are not publicly available, due to the protection of personal data, but they are available from one of the authors (POH) in response to a reasonable request.

## Abbreviations

PR: Prevalence ratio
SD: Standard deviation
BMI: Body mass index
